# A Novel Splicing Variant of *COL2A1* in a Fetus with Achondrogenesis Type II: Interpretation of Pathogenicity of In-Frame Deletions

**DOI:** 10.3390/genes12091395

**Published:** 2021-09-10

**Authors:** Valentina Bruni, Cristina Barbara Spoleti, Andrea La Barbera, Vincenzo Dattilo, Emma Colao, Carmela Votino, Emanuele Bellacchio, Nicola Perrotti, Sabrina Giglio, Rodolfo Iuliano

**Affiliations:** 1 Medical Genetics Unit, Department of Health Sciences, University “Magna Graecia” of Catanzaro, 88100 Catanzaro, Italy; valentina.bruni82@gmail.com (V.B.); cristinaspoleti@gmail.com (C.B.S.); dattilo@unicz.it (V.D.); Colaoemma@gmail.com (E.C.); perrotti@unicz.it (N.P.); 2 Medical Genetics Unit, Department of Clinical and Experimental Biomedical Sciences “Mario Serio”, University of Florence, 50121 Florence, Italy; andrea.lab84@gmail.com; 3 Fetal Medicine Unit, ASL Bari, 70012 Bari, Italy; carmela.votino@gmail.com; 4 Genetics and Rare Diseases Research Division, Bambino Gesù Children’s Hospital, IRCCS, 00165 Rome, Italy; Emanuele.Bellacchio@gmail.com; 5 Unit of Medical Genetics, Department of Medical Sciences and Public Health, University of Cagliari, 09124 Cagliari, Italy; sabrinar.giglio@unica.it

**Keywords:** skeletal dysplasia, achondrogenesis type II, *COL2A1*, splicing variant, minigene assay, in-frame deletion

## Abstract

Achondrogenesis type II (ACG2) is a lethal skeletal dysplasia caused by dominant pathogenic variants in *COL2A1*. Most of the variants found in patients with ACG2 affect the glycine residue included in the Gly-X-Y tripeptide repeat that characterizes the type II collagen helix. In this study, we reported a case of a novel splicing variant of *COL2A1* in a fetus with ACG2. An NGS analysis of fetal DNA revealed a heterozygous variant c.1267-2_1269del located in intron 20/exon 21. The variant occurred de novo since it was not detected in DNA from the blood samples of parents. We generated an appropriate minigene construct to study the effect of the variant detected. The minigene expression resulted in the synthesis of a *COL2A1* messenger RNA lacking exon 21, which generated a predicted in-frame deleted protein. Usually, in-frame deletion variants of *COL2A1* cause a phenotype such as Kniest dysplasia, which is milder than ACG2. Therefore, we propose that the size and position of an in-frame deletion in *COL2A1* may be relevant in determining the phenotype of skeletal dysplasia.

## 1. Introduction

Achondrogenesis type II (ACG2) is a rare, lethal genetic disease that is part of an uncommon skeletal dysplasia group. ACG2 is characterized by a short neck with macrocephaly; a small thorax; underdeveloped lungs; a prominent abdomen; short arms; short legs; and incomplete ossification of the vertebral column, sacrum, and pubic bones. Moreover, it is marked by distinctive facial features such as a prominent forehead; a small chin; and in some cases, a cleft palate [1].

ACG2, first described by Langer [2] and Saldino [3], is caused by a dominant variant in *COL2A1*. Pathogenic variants in this gene cause different skeletal disorders, including achondrogenesis type II and hypochondrogenesis (MIM#200610), Kniest dysplasia (MIM#156550), platyspondylic dysplasia Torrance type (MIM#151210), spondyloepiphyseal dysplasia congenita (SEDC) (MIM#183900), spondyloperipheral dysplasia (MIM#271700), SED with metatarsal shortening (formerly Czech dysplasia) (MIM#609162), SED with pronounced metaphyseal changes (including SEMD Strudwick type) (MIM#184250), and Stickler syndrome (MIM#108300) [4,5].

ACG2 is the most severe phenotype, while Kniest and SED dysplasia are milder but still have grave conditions, characterized by skeletal anomalies [6]. Stickler syndrome is the mildest condition, and frequently, ocular findings are the only clinical manifestations. Different types of *COL2A1* variants are associated with the various phenotypes [7].

*COL2A1* is located at 12q13.11 (MIM#120140) and contains 54 exons. It encodes the polypeptide subunit of the type II collagen, which is a homotrimer formed by three α-1(II) chains [8]. Similar to other collagens, a repeating Gly-X-Y motif, which is fundamental for homotrimer formation, characterizes the COL2A1 protein. In fact, in ACG2, the most common *COL2A1* variants affect the glycine residue in Gly-X-Y repeats of the α-1 chain [9,10]. These variants cause a dominant-negative effect that dramatically impairs homotrimer assembly and stability, leading to growth bone alterations and severe phenotypes such as ACG2 and hypochondrogenesis. In contrast, nonsense mutations or out-of-frame deletions can cause a premature stop codon that leads to haploinsufficiency, resulting in a decrease in collagen synthesis, as well as diseases with milder phenotypes than that observed in ACG2 [7,11]. Splice site variants leading to an in-frame deletion of *COL2A1* have already been described and are mainly associated with Kniest dysplasia [12,13]. In this study, we reported a case of a novel *COL2A1* splicing mutation in a fetus with an ACG2 phenotype, and tentatively explained this uncommon genotype–phenotype association. Moreover, we performed a minigene assay to show that the splicing variant generated an exon skipping, leading to an in-frame deletion of *COL2A1*.

## 2. Materials and Methods

### 2.1. Case Report

A 35-year-old Italian primigravida was referred to our genetic unit at 18-week gestation (WG) due to abnormal ultrasonographic findings. The parents of the fetus were non-consanguineous, and the familial history did not reveal any relevant information. Informed written consent for both diagnostic and research purposes was obtained from the parents.

**Figure 1:**
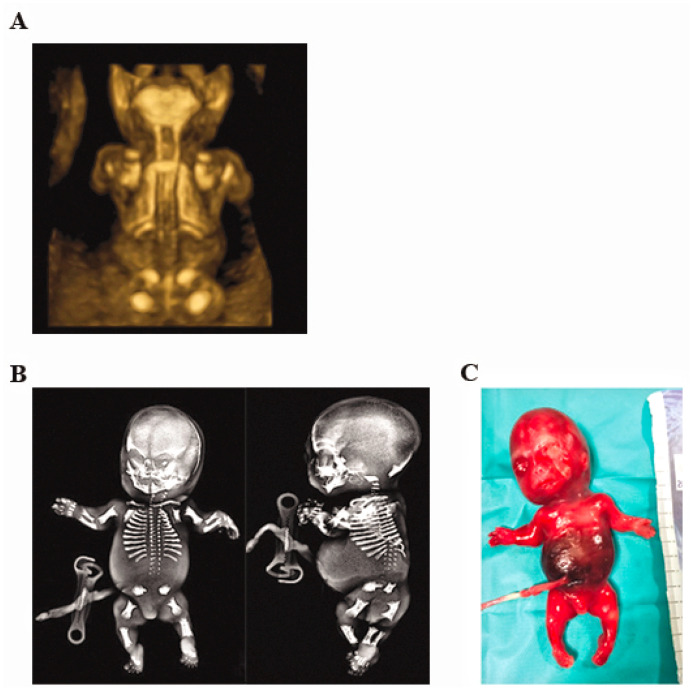
(**A**) Three-dimensional ultrasound image of the fetus showing reduced ossification of the sacrum, iliac spines, and arched femurs. (**B**) Radiograph of the fetus showing reduced ossification of the sacrum, iliac spines, and arched femurs. (**C**) Frontal view of the fetus postmortem.

A three-dimensional ultrasound was performed to examine the fetus. The fetal 3D ultrasound examination showed a brachycephaly, a prominent abdomen, short limbs (<at the third centile), a facial dysmorphism characterized by abnormal implantation of the ears, microretrognathia, frontal bossing, a long philtrum, and congenital megalophthalmos. The three-dimensional rendering of the fetal skeleton showed reduced ossification of the sacrum, iliac spines, and arched femurs (Figure 1A). Amniocentesis performed at 19-WG for fetal karyotyping and array-CGH were negative.

At 20-WG gestation, the parents decided to interrupt the pregnancy. Postmortem radiography confirmed reduced ossification of the sacrum and arched femurs (Figure 1B). An autopsy was also performed (Figure 1C), and a cleft palate was observed. The prenatal and postnatal findings were strongly suggestive of skeletal dysplasia, in particular, of achondrogenesis type 2.

### 2.2. Gene Sequencing

Whole exome sequencing (WES) and in silico analysis of *COL2A1* (NM_001844.5) on the fetal DNA revealed a heterozygous base-pair deletion involving the exon 21 of the *COL2A1* gene (c.1267-2_1269del).

DNA from the fetal tissue was enriched with SeqCap EZ Exome v3, prepared according to the manufacturer’s protocol (Nimblegen, Roche, Basel, Switzerland), and 150 × 2 bp end sequenced on NextSeq550 (Illumina Inc., San Diego, CA, USA) with a 100× mean coverage. Reads were aligned with the human genome reference sequence (hg19) using Burrows–Wheeler Aligner (BWA) and then mapped and analyzed by the IGV software (Integrative Genome Viewer, 2013 Broad Institute, Cambridge, MA, USA). The variant call for identifying nucleotide variants was performed using automated in-house pipelines (Genome Analysis ToolKit Unified Genotyper Module, GATK, Cambridge, MA, USA). Variants were classified following the guidelines of the American College of Medical Genetics and Genomics (ACMG) [14]. The data interpretation was made by in silico filtering for *COL2A1,* and a heterozygous base-pair deletion involving the exon 21 of *COL2A1* (c.1267-2_1269del) was detected.

Genomic DNA was also extracted from the blood samples of the patient’s parents. The genomic DNA was amplified by the polymerase chain reaction (PCR) with primers forward 5′-ggttggggttcattctttgc-3′ and reverse 5′-tgatggggtttgactccaga-3′ for exon 21 of *COL2A1*. The PCR products were then purified and bidirectionally sequenced on a 310 ABI PRISM Genetic Analyzer (Applied Biosystems, Waltham, MA, USA). The c.1267-2_1269del variant of *COL2A1* was confirmed in the fetus and excluded in the parents.

### 2.3. Minigene Assay

A wild-type DNA fragment containing *COL2A1* from exon 19 to exon 22 was amplified by PCR using human genomic DNA as a template and oligonucleotides, including restriction sites for XhoI and HindIII: Forward 5′-cagAAGCTTgggttgggtgcatgtgcataat-3′ and reverse 5′-gtaCTCGAGtgaagctgtatctgggccttct-3′. The wild-type fragment was then used as a template to generate a fragment containing the variant c.1267-2_1269del by site-directed mutagenesis (overlap extension PCR) using the following additional oligonucleotides: Forward 5′-tcatgcccacgctcctggcatt-3′ and reverse 5′-aatgccaggagcgtgggcatga-3′. All the PCR amplifications were performed with the Pfu DNA polymerase. The wild-type and mutated amplified products were purified by a gel extraction kit (QIAquick Gel Extraction kit, Qiagen, Hilden, Germany) and then cloned into a pcDNA3 expression vector at the XhoI and HindIII restriction sites to generate two minigene constructs: COL2A1-WT and COL2A1-MUT. Recombinant vectors were checked by a sequence analysis.

Human embryonic kidney fibroblasts (HEK 293T) cells were grown in Dulbecco’s modified Eagle’s medium, DMEM (Sigma-Aldrich, St. Louis, MO, USA), supplemented with 10% fetal bovine serum at 37 °C with 5% CO_2_ atmosphere. Transient transfection was performed on 70% confluent cells plated in six-well dishes with 2 μg of wild-type or mutant minigenes, using the Lipofectamine 3000 transfection reagent (Thermo Fisher, Waltham, MA, USA), according to the manufacturer’s instructions. The cells were harvested 48 h after the transfection, and total RNA was extracted using the RNeasy Mini Kit (Qiagen, Hilden, Germany) by following the manufacturer’s instructions. Total RNA samples were reverse-transcribed to cDNA using the High-Capacity RNA-to-cDNA Kit (Applied Biosystems). The cDNA was amplified by PCR using the following primers: Forward 5′-tcgcggtgaacctggtact-3′ (sequence located in exon 19 of *COL2A1*) and reverse 5′-gccttgttcacctttgaagcca-3′ (sequence located in exon 22 of *COL2A1*). PCR products were visualized by 1.5% agarose gel, and the cDNA amplified fragments were purified by a gel extraction kit (QIAquick Gel Extraction kit, Qiagen, Hilden, Germany) and then bidirectionally sequenced.

## 3. Results

**Figure 2:**
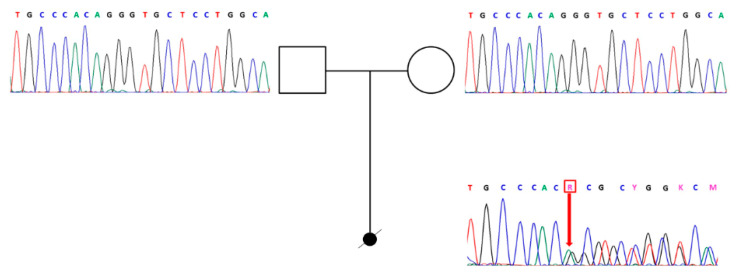
Genealogic tree of the family with Sanger sequencing of exon 21 of *COL2A1*.

WES of the fetal DNA revealed a heterozygous variant c.1267-2_1269del (NC_000012.11: g.48380957_48380961del) located in intron 20/exon 21 of *COL2A1* (NM_001844.5). This variant has never been reported in control populations (dbSNPs, EVS, GnomAD, and 1000 genomes databases) and, as a pathogenic variant, in ClinVar and LOVD databases. Sanger sequencing across the site of the variant observed in the affected fetus confirmed the presence of the wild-type sequence in the unaffected parents, consistent with a de novo mutation in the fetus (Figure 2).

*COL2A1* in-frame deletions have already been reported and are responsible for a milder phenotype. We considered this variant as strongly causative of the observed phenotype since we did not identify additional variants in *COL2A1* coding and regulatory regions. In addition, we did not detect rare homozygous or putative compound heterozygous combinations in other genes previously reported in overlapping conditions.

**Figure 3:**
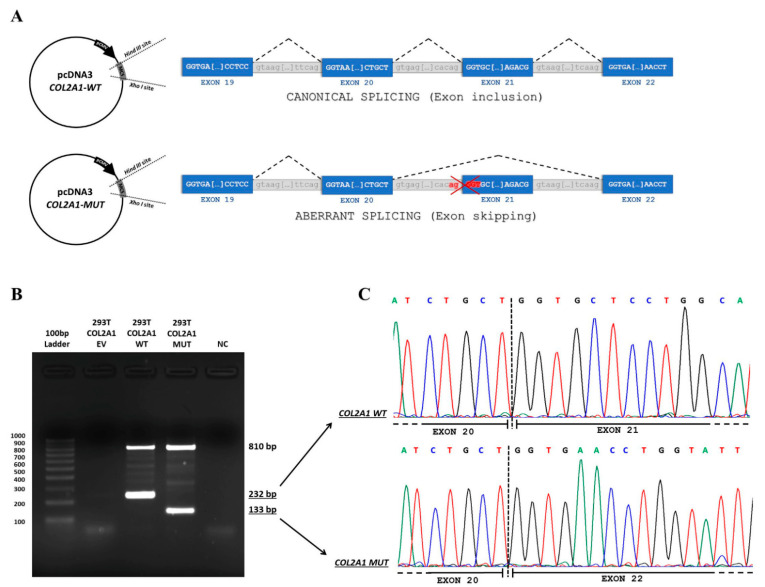
(**A**) Schematic representation of minigene construct. Exons 19, 20, 21, and 22 are represented, and expected spliced forms are indicated on the right side. Wild-type and mutated constructs are represented on the top and the bottom, respectively. (**B**) Electrophoresis of PCR amplification of nucleic acids extracted from HEK293T-transfected cells. RNA samples were reverse-transcribed and subjected to PCR amplification with specific primers located in exons 20 (Forward) and 23 (Reverse) of *COL2A1*. The DNA band of 810 bp represents the non-spliced DNA of the transfected constructs, while the 232 and 133 bp bands represent the spliced transcripts in the cells transfected with the wild-type and mutated constructs. DNA was purified from the 232 and 133 bp bands and then sequenced (EV: Empty vector; WT: Wild-type minigene construct; MUT: Mutated minigene construct; and NC: Negative control). (**C**) Electropherograms of amplified products (wild-type and mutated) are shown in panel B.

Since the variant does not contain the splice acceptor site of exon 21 of *COL2A1*, to study the variant’s effect, we generated an appropriate minigene construct, as described in the Methods section (Figure 3A). We transfected mutant and wild-type minigene constructs in HEK293T cells and detected the expression of minigenes by RT-PCR. The band of amplified cDNA corresponding to the spliced transcript in the mutant sample was lower than that detected in the wild-type sample, which is consistent with the skipping of exon 21 in *COL2A1* messenger RNA (Figure 3B). Furthermore, the sequence of cDNA-amplified products confirmed that, in the mutant sample, the minigene expression resulted in the skipping of exon 21 (Figure 3C). The skipping of exon 21 generated an in-frame deletion (p.Gly423_Thr455del) in the COL2A1 protein (NP_001835.3). The PROVEAN algorithm predicted this variant to be deleterious [15], with a score of −122.637 recorded (cut-off of −2.5).

## 4. Discussion

To classify the detected in-frame deletion as pathogenic, we applied the following ACMG criteria: PS2 (de novo variant), PM4_Strong (protein changing length variant), PM2 (absent in population databases), PP3 (computational evidence), and PP4 (patient’s phenotype highly specific for a gene). The minigene assay demonstrated that the splicing variant generated an in-frame deletion, and therefore, we applied the PM4 criterion. In addition, we promoted PM4 to a strong criterion since in-frame deletions in *COL2A1* are likely to be pathogenic [7,9].

*COL2A1* pathogenic variants are associated with many skeletal dysplasia, with a series of phenotypes ranging from lethal to relatively mild forms often reported [6]. Since a dominant inheritance characterizes the diseases associated with *COL2A1* mutations, two mechanisms have been linked with pathogenetic *COL2A1* variants: Haploinsufficiency and negative dominance, with the latter being related to a more severe phenotype [9].

The substitution of glycine in the G-X-Y repeat of COL2A1 protein is the typical example of dominant-negative mutation and is mainly associated with ACG2. In contrast, variants leading to a premature stop codon can activate a mechanism of nonsense-mediated decay (NMD), resulting in haploinsufficiency of *COL2A1*. These variants are mainly associated with the mildest clinical forms of skeletal dysplasia [7,9].

Variants that affect splicing can generate either an out-of-frame protein, possibly activating an NMD mechanism or an in-frame protein with a small deletion. The in-frame deletions of *COL2A1* are primarily associated with Kniest dysplasia [12], an intermediate phenotype that is less severe than ACG2. In contrast, a milder phenotype is expected in the case of NMD activation caused by an out-of-frame exon deletion [9]. Functional studies assessing the pathogenicity of the variant are thus required in order to determine whether a new variant in a splice site generates a frameshift [16]. In our case, the detected splice site variant generated an in-frame deletion that showed a substantial expression in a minigene splicing assay. Therefore, the severe phenotype observed was possibly due to a dominant-negative mechanism caused by the resulting protein deletion variant.

**Figure 4:**
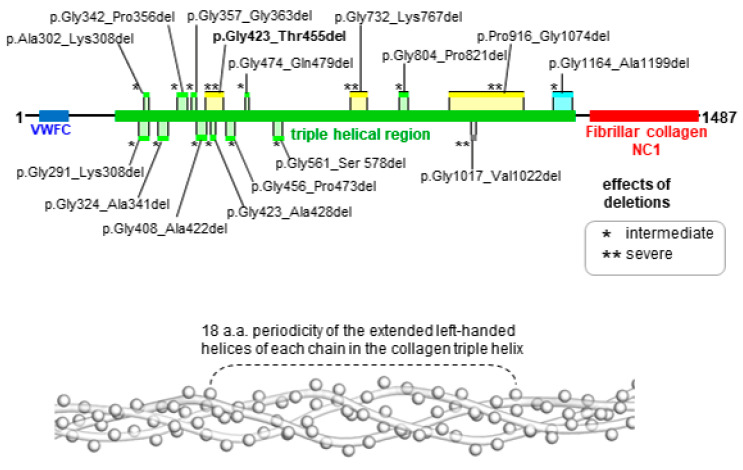
Schematic view of collagen α-1(II) chain (top) that shows the novel protein deletion position reported in this work and previously published deletions. Deletions, characterized by the loss of 18 amino acids or fewer, causing intermediate (*) and severe (**) effects are shown in light green and grey, respectively. Larger deletions causing intermediate (*) and severe (**) effects are shown in light blue and yellow, respectively. In the severe group, there are cases of ACG2 or hypochondrogenesis. In the intermediate group, there are cases of Kniest dysplasia or SEDC. Periodicity of 18 amino acids (bottom) of the extended left-handed helix of each protein chain in the collagen triple helix are exemplified here by a portion of the Protein Data Bank structure 3HQV (C^α^ atoms are represented by spheres). Amino acid numbering refers to the protein with NCBI accession code NP_001835.3. **p.Gly423_Thr455del** (our study); p.Gly732_Arg767del and p.Gly1017_Val1022del (Körkkö et al. 2000) [18]; p.Pro916_Gly1074del (Barat-Houari et al. 2016) [10]; p.Gly291_Lys308del (Winterpacht et al. 1993) [19]; p.Ala302_Lys308del (Bogaert et al. 1994) [20]; p.Gly324_Ala341del, p.Gly357_Gly363del, p.Gly423_Ala428del, and p.Gly456_Pro473del (Wilkin et al. 1999) [12]; p.Gly342_Pro356del (Fernandes et al. 1988; Wada et al. 2011) [21,22]; p.Gly408_Ala422del (Spranger et al. 1997) [23]; p.Gly474_Gln479del (Winterpacht et al. 1994) [24]; p.Gly561_Ser578del (Weis et al. 1998) [13]; p.Gly804_Pro821del (Mortier et al. 2000) [25]; p.Gly1164_Ala1199del (Lee et al. 1989) [26].

To better understand the phenotype generated by the *COL2A1* variant detected by us, we listed variants of *COL2A1* already reported in the literature to cause in-frame deletions in order to perform a genotype–phenotype correlation. The size of the in-frame deletion seems to be very important for the determination of the phenotype. Variants with deletions of 18 amino acids or fewer are associated with less severe phenotypes (Figure 4). Notably, 18 amino acids correspond to the number of residues in a turn of the extended left-handed helix formed by each collagen chain in the collagen triple helix (Figure 4) [17].

Therefore, a deletion longer than 18 amino acid residues might cause the formation of more extended loops, introducing bulkier structural defects in the heterotrimers, whereas shorter loops might be accommodated more easily, producing less irregular protein assemblies. There are only two exceptions to this rule: the quite long deletion p.Gly1164_Ala1199del at C-termini associated with an intermediate phenotype [26], and the small deletion p.Gly1017_Val1022del associated with a severe phenotype [18]. However, the p.Gly1164_Ala1199del variant occurs at the C-terminal side of the triple-helical region. In this location, it might simply disfavor the initial process of triple helix formation. Hence, producing less aberrant multimers and reducing dominant effects. Therefore, the unique exception remains the p.Gly1017_Val1022del variant [18]. In a large study that attempted to show a genotype-phenotype correlation for *COL2A1*-related disorders, severe phenotypes were found to be more associated with in-frame deletions than frameshift variants [7].

In vitro studies have shown that the in-frame deletion of *COL2A1* generates a loop structure in the trimer of collagen type II molecules that are highly susceptible to trypsin’s proteolytic activity [13]. In addition, heterotrimers composed of mutant and wild-type sequences assemble with local disrupted domains at the deletion site, destabilizing the collagen fiber structure. Therefore, a more extended deletion could more efficiently destabilize the trimer, increasing the proteolytic damage operated by matrix proteases.

In the two studies, transgenic mice were generated with a construct containing a human *COL2A1* with an in-frame deletion. In the first study, exon 7 was eliminated, while in the second one, 12 exons were removed [27,28]. These transgenic mice showed severe chondrodysplasia and died shortly after birth. Vandenberg et al. [27] hypothesized a mechanism of collagen suicide induced by the short deleted COL2A1 protein. Furthermore, in both cases, a high expression of the transgene seems also relevant to determine the phenotype. Therefore, the expression of a *COL2A1* pathogenic variant could determine the generation of a product that interferes with the wild-type form, inhibiting the correct formation of collagen trimers. In our case, the minigene assay was valuable for defining the expression of the *COL2A1* carrying the splicing variant.

In conclusion, our study showed that the size and position of the deletions across the helical regions could be relevant for determining the pathogenicity of *COL2A1* variants capable of generating in-frame deletions. More cases involving large in-frame deletions are needed to confirm this hypothesis. Functional studies are critical to demonstrate the generation of an expressed in-frame deletion variant, which could help variant classification or explain variant pathogenicity.
